# Assessing Disparities in Who Accepts an Early Palliative Care Consultation

**DOI:** 10.3390/curroncol32090485

**Published:** 2025-08-30

**Authors:** Heather Halperin, Philip Akude, Seema King, Patricia Biondo, Aynharan Sinnarajah, Desiree Hao, Jessica Simon

**Affiliations:** 1 Department of Internal Medicine, University of Calgary, Calgary, AB T2N 1N4, Canada; 2 Department of Oncology, Division of Palliative Medicine, University of Calgary, Calgary, AB T2N 5G2, Canada; philip.akude@ucalgary.ca (P.A.);; 3 Department of Oncology, Section of Medical Oncology, Arthur JE Child Comprehensive Cancer Centre, Calgary, AB T2N 5G2, Canada

**Keywords:** health equity, referral and consultation, Carcinoma, non-small-cell lung

## Abstract

Early palliative care improves quality of life for patients with life-limiting illnesses, but access is often inequitable. The goal of this study was to assess disparities in early specialist palliative care (SPC) consultation among newly diagnosed stage IV lung cancer patients. All newly diagnosed stage IV lung cancer patients in southern Alberta, Canada (June 2021–March 2022) were offered SPC consultations from a multidisciplinary team, post-oncology visit. A retrospective chart review analyzed demographic factors and consultation outcomes (accepted, ineligible, declined/unreachable), using the Pampalon Deprivation Index and NamSor surname analysis as proxies for equity-related variables. Of 113 patients, 76.2% were eligible for consultation, and 67.4% of those accepted consultation. Older age (>65 years), male sex, and high deprivation were linked to declining SPC (*p* < 0.05–0.01). Conversely, living alone or with a non-partner increased acceptance (*p* < 0.05). Age, sex, deprivation, and living situation influenced SPC acceptance. Identifying disparities can guide interventions to improve equitable access.

## Simple Summary

Early palliative care can improve quality of life for people with advanced cancer, but not everyone accesses it equally. In this study, all patients newly diagnosed with metastatic lung cancer in southern Alberta were offered a home palliative care consultation after their first oncology appointment. We looked at which patients accepted, declined, or were unreachable, and explored factors such as age, sex, living situation, and social or economic challenges. We found that older adults, men, and those facing more social deprivation were more likely to decline care, while people living alone or with someone other than a partner were more likely to accept it. These findings show that even when care is offered to everyone, some groups are still less likely to receive it. Understanding these patterns helps us design better approaches to ensure all patients who need palliative care can access it fairly.

## 1. Introduction

The quintuple aims of healthcare include achieving health equity, defined as “the absence of avoidable or remediable differences among groups of people,” and is achieved when everyone can attain their full health potential, where no one is disadvantaged due to social position or other socially determined circumstances [1,2]. Providers are called upon to “…identify disparities, design and implement evidence-based interventions to reduce them, invest in equity measurement, and incentivize the achievement of equity” [1].

Access to palliative care is a World Health Organization recognized right that should be available to all, regardless of age, sex or gender, illness trajectory, community, socio-economic status (SES), or ethnicity [3,4]. Unfortunately, this essential care is not equitably distributed or accessed [5,6,7]. Multiple intersecting factors—patient, clinician, and systemic—affect palliative care use [8,9,10], including an effective offer of support and patient choice in the uptake of that offer.

Our Palliative Care Early and Systematic (PaCES) project team of patients, clinicians, and researchers codesigned, evaluated and implemented evidence-based practices to increase equitable uptake of timely palliative care [11]. To promote universal access, a palliative care nurse called all people newly diagnosed with stage IV lung cancer, after first oncologist appointment, offering a specialized palliative care consultation (SPC) [12,13]. Prospective evaluation found high patient-rated acceptability for both the calls and consults with no significant differences across age, education, income, second language, or ethnicity [13]. However, sampling bias or patient and caregiver experience may mean these results were not fully representative of all equity-deserving groups in the local population [13,14,15].

Thus, the objective of this study was to assess if there were measurable inequities amongst all patients who were *offered* SPC consultation and to explore patient differences in *uptake* between those who accepted, declined, were unreachable, or were already receiving palliative care. Inspired by the quintuple aim, this analysis asks: has “universal access” truly been achieved?

## 2. Materials and Methods

**Figure 1:**
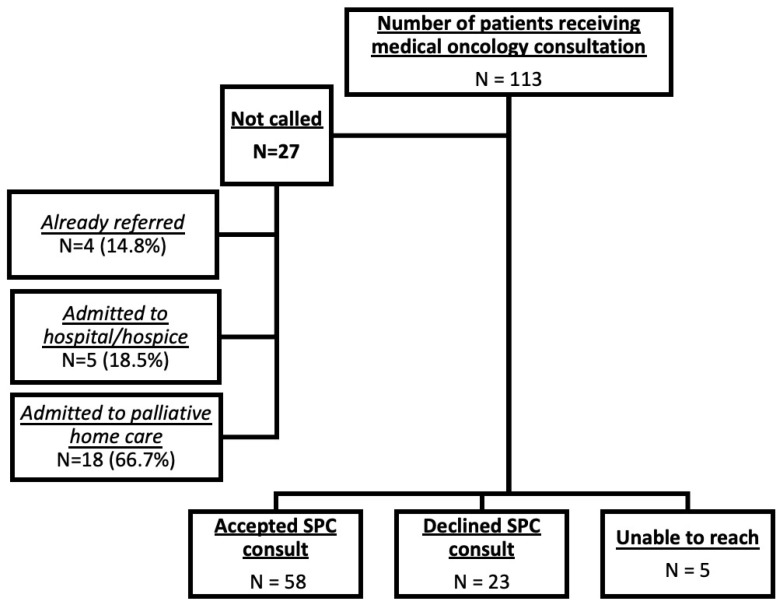
Breakdown of patients not called, accepting of palliative care consultation, declined consultation, and patients who were unable to be reached.

This retrospective, secondary cohort analysis included all patients with stage IV lung cancer in southern Alberta, Canada from June 2021 to March 2022 who completed their first out-patient medical oncologist visit. Two SPC nurses reviewed weekly outpatient lung cancer clinic lists to identify new patients with stage IV non-small cell lung cancer and contacted all eligible patients or their caregivers to offer an in-home SPC consultation within one to two weeks of their first oncology visit, unless the patient was ineligible for the call (had already received SPC consultation, was receiving homecare, hospitalized, or had died) (Figure 1). Further details of accrual are described by King et al. in the prospective study by our group [13].

Demographic data related to equity factors—age, gender, immigration status, living arrangement (lives alone, with partner, or with non-partner), and consultation outcome (accepted, declined/unreachable, ineligible for call)—were extracted by one researcher (HH). Ethnicity was extrapolated using Namsor™ Applied Onomastics Paris, Ile-de-France, France Reg. 81148844400019–VAT FR84811488444, which analyzes surnames via machine learning of historical census data [16,17,18]. Independently, deprivation level was estimated using the Pampalon Deprivation Index (Québec, QC, Canada), a census-based measure of income, education, and employment by postcode, providing an overall measure of material and social deprivation [19,20,21]. Higher Pampalon Index scores indicate a higher level of deprivation, which can be associated with health disparities and poorer health outcomes [21,22,23].

Descriptive statistics (chi-squared, Fisher exact tests) examined univariate significance between equity factors and three consultation outcomes (accepted, declined/unreachable after three phone call attempts, ineligible for call). We estimated an ordered probit model to evaluate the relationship between the ordered outcome variables—booked consultation, ineligible, and declined/unreached by phone—and the collected independent variables. Using the regression analysis, considering relationships between outcomes and the collected equity factors, analysis was based on the changes in the probability of outcome because of variations in equity factors.

This study was approved by the Health Research Ethics Board of Alberta-Cancer Committee (HREBA.CC-21-0026).

## 3. Results

### 3.1. Descriptive Statistics

During the 9-month study, 113 patients were diagnosed. Of 86 patients called, 81 were reached by phone; 67.4% (*n* = 58) accepted consultation, while 26.7% (*n* = 23) declined consultation. The details are shown in Figure 1.

### 3.2. Univariate Analysis

Comparison of the three groups (those who accepted the consultation, declined the consultation/were not reached, and ineligible for call), revealed a statistically significant difference in age (*p* = 0.01), with fewer aged under 65 years in the group being ineligible for call (Table 1). No other statistically significant differences were identified for other equity factors (sex, immigration status, living arrangement, ethnicity, or deprivation index).

### 3.3. Regression Analysis

**Figure 2:**
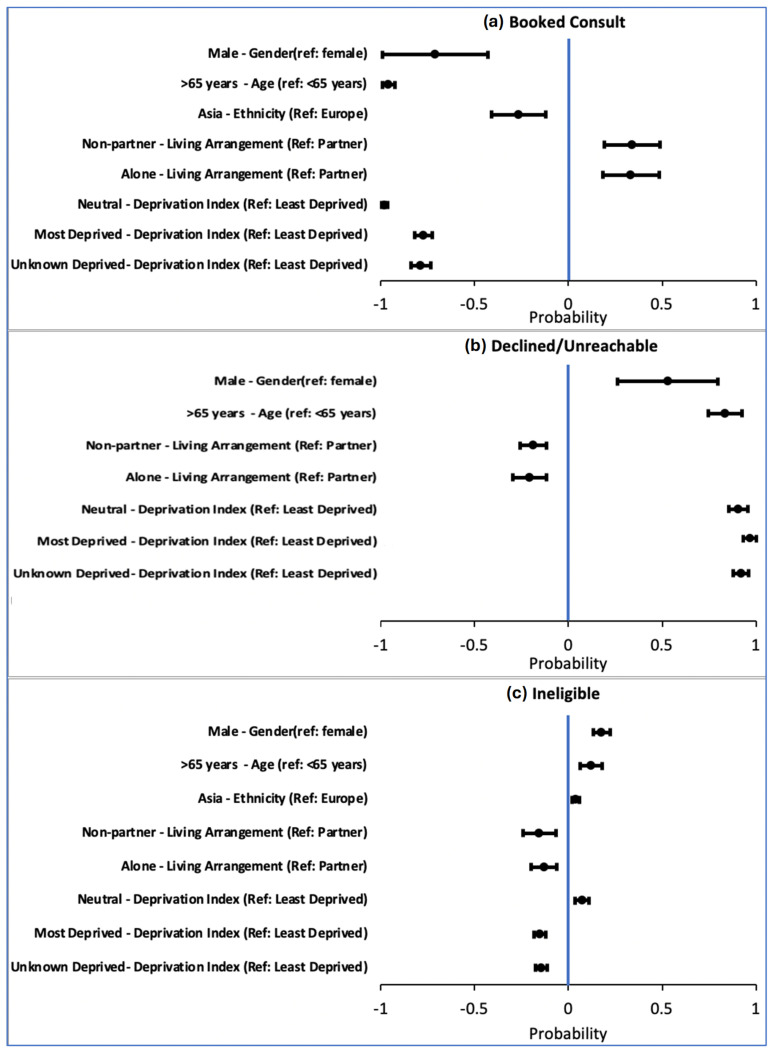
Forest plots displaying impact of statistically significant demographics by regression analysis on probability of individual outcome (**a**) booked consultation, (**b**) declined/unreachable, or (**c**) ineligible. Negative probability is associated with less likely outcome, whereas positive probability is associated with more likely outcome.

From the regression analysis, several equity factors were associated with probability of declining consultation (Figure 2). Males (53%, *p* < 0.05, ref: female), age > 65 years (84%, *p* < 0.01, ref: age < 65 years), and patients who were more deprived (92%, *p* < 0.01, ref: least deprived) were more likely to decline palliative consultation. Conversely, those living with a non-partner (34%, *p* < 0.05) or alone (33%, *p* < 0.05) were more likely to accept palliative care consultation. This may reflect that individuals living alone or with non-partners have fewer informal caregiving supports and may be more open to receiving formal healthcare services.

## 4. Discussion

Providing a universal telephone offer of a timely palliative care consultation appears to have reached all equity groups but patient uptake of the offer differed. Patients who declined the offer of a consultation early were more likely to be living with factors associated with deprivation, including older age, male sex, and being more socio-materially deprived. It is important to emphasize that declining a consultation may reflect individual preference or self-perceived lack of need, rather than an inequity. However, understanding patterns in who declines care can help us identify whether certain groups are being systematically underserved, even when access is offered. Previous studies similarly report patients experiencing health inequities, including older adults, males, and socioeconomically disadvantaged people, are less likely to utilize palliative care services [24,25,26,27].

In our cohort, older patients were less likely to accept an early palliative care consultation, a pattern that aligns with prior research. Cancer patients above 65 years were observed by Saeed et al. to be less likely to prefer palliative care [28]. Further, a recent systematic review found that older age is consistently associated with reduced referral to and uptake of palliative care among patients with cancer [29]. Qualitative studies suggest that older adults may decline palliative care due to concerns about stigma, the perception that such services signal a transition away from active treatment, and the belief that referral is premature [27]. Together, these findings highlight how age-related attitudes and expectations can shape engagement with palliative care and may help explain the lower acceptance observed in our study.

Evidence on gender-based referral patterns to palliative care is mixed [26]. Some studies reported women were more likely to be referred to palliative care services [26,30,31]. A plausible hypothesis for this includes gendered caregiving roles contributing to differences in healthcare utilization, whereby men may rely more on informal care from female household members, while women, less likely to have equivalent informal support, may need to use more formal healthcare services [32]. Broader literature observes women generally use healthcare more frequently, including primary care, possibly contributing to our finding that males were more likely to decline the consult offer [33,34].

While the impact of SES on palliative care uptake is complex, trends show that those with fewer SES barriers access care more often [35]. This has been hypothesized, at least in part, to patients with lower SES having decreased awareness of palliative care services, lower referral rates, and different priorities [36]. Our findings were identified in a publicly funded, Canadian, healthcare system, where access to care is not directly constrained by individual financial capacity. Socioeconomic disparities in health outcomes may be exacerbated in systems where healthcare incurs direct costs, potentially amplifying barriers to care for lower-income populations [37].

In our study, patients with low social capital (living without a partner or alone) were more likely to accept palliative care consultation compared to patients with a partner. This contrasts with some previous studies, that have found patients who were not married (i.e., lower social capital) to be less likely to access palliative care services [26,38,39,40]. This finding is particularly interesting when contrasted with the negative association between social deprivation and acceptance. Living alone may increase openness to support due to reduced informal caregiving networks, while deprivation might hinder access due to mistrust, lower health literacy, or other systemic barriers, suggesting different mechanisms may be at play. For instance, a study from the Danish Palliative Care Database found that cohabiting individuals were more likely to be admitted to specialized palliative care compared to those who were widowed [41]. Another study using the National Mortality Followback Survey found that unmarried individuals were more likely to live their last year of life and die in nursing homes, and less likely to receive formal or informal care, compared to married individuals [42].

Patients who were ineligible for the call (due to prior palliative care, hospitalization, or death) were more likely to be older, male, or of Asian descent. While this may reflect effective identification of those in need earlier in the disease trajectory, it may also suggest that certain groups are diagnosed later, with more aggressive disease, or at more advanced stages, which could point to broader systemic disparities. However, this interpretation is speculative and warrants further investigation.

### Strengths and Limitations

Measuring potential equity factors associated with uptake of palliative care is an important part of the quintuple aim of heath care. However, the retrospective design limits our ability to assess undocumented equity factors. SES and ethnicity have not been routinely collected in our healthcare system and could only be estimated using the proxy of postal code and last name [43]. We recognize that using these proxies—especially name-based ethnicity estimates—can introduce risk of misclassification and may be perceived as discriminatory. This points to the on-going need for better health system collection of self-identified race and other identity factors [44]. Without other important “PROGRESS-Plus” characteristics that stratify health opportunities and outcomes being recorded (such as occupation, religion, education, sexuality and personal characteristics associated with discrimination), other important sources of inequity in palliative care utilization may continue to be missed [45].

More work is now needed to explore why patients decline consultations and how equity barriers can be addressed at the point of offer. Additionally, our findings may be specific to the context of an in-home consultation. It is possible that if palliative care had been offered as a hospital- or clinic-based service, the uptake patterns would have differed. Future research should investigate whether the setting of offer affects acceptance. Understanding motivations, whether grounded in fear, mistrust, lack of perceived need, or cultural attitudes, is crucial to designing interventions that are respectful of patient autonomy while addressing avoidable disparities. Previous reviews, such as that by Jones et al., revealed that targeted interventions can improve palliative outcomes for underrepresented groups [46].

## 5. Conclusions

By identifying the inequities in age, gender, and material deprivation in those who accepted or declined the early offer of a supportive and palliative care consultation, we are motivated to develop more tailored interventions to increase uptake of palliative care supports for all who could benefit. Our study also highlights the need for more health equity factors to be proactively assessed and documented in health records to enable identification and to address inequities. By measuring the inequities that exist within our healthcare systems, we may better embark on solutions.

## Figures and Tables

**Table 1:** Equity factors, divided by accepted and declined consultation/unable to reach. * Statistical significance between health equity factors and outcome; *p*-values are from chi-squared and fisher exact tests, as appropriate.

		Outcome			*p*-Value
		Consult Booked	Declined/Unreachable	Not Called	
Gender					0.09
	Female	32 (55%)	10 (36%)	9 (33%)	
	Male	26 (45%)	18 (64%)	18 (67%)	
Age					0.01 *
	Under 65	23 (40%)	12 (43%)	3 (11%)	
	65 and Over	35 (60%)	16 (57%)	24 (89%)	
Immigration Status					0.48
	Canadian	34 (59%)	19 (68%)	12 (44%)	
	Not Canadian	17 (29%)	7 (25%)	10 (37%)	
	n/a	7 (12%)	2 (7%)	5 (19%)	
Living Arrangements					0.53
	Partner	38 (66%)	18 (64%)	12 (44%)	
	Non-partner	5 (9%)	1 (4%)	4 (14)	
	Alone	12 (21%)	7 (25%)	9 (33%)	
	n/a	3 (5%)	2 (7%)	2 (7%)	
Ethnicity					0.78
	Europe	44 (76%)	20 (71%)	21 (78%)	
	Asia	8 (14%)	6 (21%)	5 (18%)	
	Africa	6 (10%)	2 (7%)	1 (4%)	
Deprivation Index					0.74
	Most deprived	5 (9%)	2 (7%)	1 (4%)	
	Neutral	33 (57%)	17 (61%)	14 (52%)	
	Least Deprived	8 (14%)	4 (14%)	8 (30%)	
	Unknown	12 (21%)	5 (18%)	4 (15%)	

## Data Availability

Aggregate, de-identified data may be made available on reasonable request from the corresponding author. The data are not publicly available, consistent with ethics approval.

## Abbreviations

The following abbreviations are used in this manuscript:

SPC	Specialist Palliative Care
SES	Socio-Economic Status
PaCES	Palliative Care Early and Systematic (project)
PROGRESS-Plus	Place of residence, Race/ethnicity, Occupation, Gender, Religion, Education, Socioeconomic status, Social capital, and additional context-specific factors (Plus)
